# Three-dimensional mapping of tick-borne encephalitis virus distribution in the mouse brain using a newly engineered TurboGFP reporter virus

**DOI:** 10.1080/22221751.2025.2542246

**Published:** 2025-07-31

**Authors:** Michaela Berankova, Simone Leoni, Jiri Holoubek, Jan Haviernik, Jiri Salat, Denis Grandgirard, Stephen L. Leib, Daniel Ruzek

**Affiliations:** a Department of Experimental Biology, Faculty of Science, Masaryk University, Brno, Czech Republic; b Laboratory of Arbovirology, Institute of Parasitology, Biology Centre of the Czech Academy of Sciences, Ceske Budejovice, Czech Republic; c Laboratory of Emerging Viral Diseases, Veterinary Research Institute, Brno, Czech Republic; d Institute for Infectious Diseases, University of Bern, Bern, Switzerland; e Multidisciplinary Center for Infectious Diseases, University of Bern, Bern, Switzerland; f Graduate School for Cellular and Biomedical Sciences, University of Bern, Bern, Switzerland

**Keywords:** TBEV, reporter viruses, neurotropism, organotypic cerebellar slices, light-sheet microscopy, tissue clearing

## Abstract

Tick-borne encephalitis virus (TBEV) is a neurotropic orthoflavivirus that invades the central nervous system, leading to severe neurological manifestations. In this study, we developed a reporter virus comprising TurboGFP-expressing TBEV (tGFP-TBEV) as a versatile tool for advancing TBEV research. The tGFP-TBEV facilitates quantitative measurement of viral replication, enables precise tracking of individual infected cells, and supports high-throughput screening of potential antiviral compounds and virus-neutralization assays. Furthermore, tGFP-TBEV proved effective as a model for studying TBEV infection in rat organotypic cerebellar slices cultured *ex vivo* and for visualizing TBEV infection in the mouse brain. Using tissue-clearing protocols and light-sheet fluorescence microscopy, we achieved high-resolution, three-dimensional mapping of the TBEV distribution in the mouse brain. This analysis uncovered distinct patterns of TBEV tropism, with infections concentrated in regions associated with neurogenesis, olfactory processing, and specific neuroanatomical pathways. The ability to visualize infection at both the cellular and whole-organ level provides a new tool for detailed investigations into viral tropism, replication, and interactions with host tissues, paving the way for deeper insights into TBEV biology and the pathogenesis of tick-borne encephalitis.

## GRAPHICAL ABSTRACT

**Figure:**
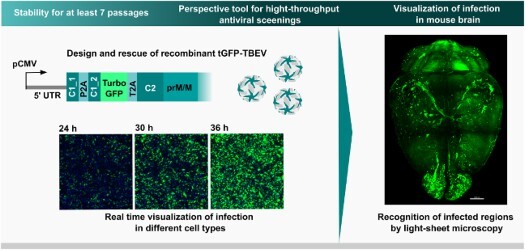

## Introduction

Tick-borne encephalitis virus (TBEV) is a neurotropic orthoflavivirus (family *Flaviviridae*, genus *Orthoflavivirus*) [1] that causes tick-borne encephalitis (TBE), a potentially life-threatening disease affecting humans and animals in endemic regions of Europe and Asia. More than 10,000 human clinical cases of TBE are reported every year [2,3]. The virus is transmitted primarily through the bite of an infected tick, with initial replication occurring in the skin and draining lymph nodes before systemic dissemination and possible invasion of the central nervous system (CNS) [4]. Within the CNS, TBEV predominantly targets neurons, leading to neuronal damage and inflammation. The infection manifests as meningitis, meningoencephalitis, and/or encephalomyelitis and can, in severe cases, lead to paralysis or death [5,6]. Moreover, a significant proportion of surviving patients experience long-lasting or permanent sequelae following infection, including fatigue, concentration difficulties, general weakness, myalgia, and sleep disturbances. These persistent symptoms can substantially impact quality of life [6-8]. Although an effective vaccine against TBE is available [9], it has certain limitations, including the necessity for regular booster doses to maintain immunity. In addition, in many cases, the financial burden is placed on the individual, which may deter people from seeking vaccination [9]. As a result, vaccination coverage remains relatively low in several endemic regions, leaving a significant proportion of the population at risk of infection [9]. Despite the severity of TBE, the mechanisms underlying TBEV neuroinvasion, neuronal tropism, and brain-specific pathogenesis remain insufficiently understood [4,10].

Animal models, particularly rodents, are essential for studying TBEV pathogenesis and host-virus interactions *in vivo*, as they recapitulate the clinical course of severe TBE in humans [8,11-13]. Additionally, mouse models that mimic subclinical and mild forms of the disease have also been developed [14,15]. However, traditional methods for investigating the viral distribution in the brain often rely on labour-intensive techniques, such as immunohistochemistry or RNA in situ hybridization, which provide limited spatial resolution and are unable to capture three-dimensional (3D) distributions. Moreover, a deeper understanding of viral dynamics in the brain requires tools that allow precise visualization of infection at the cellular level. Recently, a technique for visualizing whole-brain viral infections was developed using optical projection tomography (OPT) simultaneously with *ex vivo* MRI [10]. This method, which utilizes Langat virus, a close relative of TBEV, and *Ifnar^-/ –^* mice, offers valuable insights into viral distribution within the brain [10]. However, it requires labour-intensive procedures, including the preparation of samples and the use of large quantities of virus-specific antibodies to identify infected cells [10]. These challenges could potentially be overcome by using reporter viruses, which allow direct visualization of infection without requiring antibody-based staining.

To address these challenges, we developed a novel reporter virus, TurboGFP-expressing TBEV (tGFP-TBEV), which was engineered to express a stable fluorescent protein that facilitates real-time visualization of infected cells and/or tracking of the infection in more complex tissues without requiring secondary staining procedures. Building on previous efforts to create reporter orthoflaviviruses [16-30], including our previous first-generation reporter mCherry-TBEV [31,32], the tGFP-TBEV construct incorporates optimized genetic elements for enhanced stability and fluorescence intensity, enabling robust detection of viral replication in diverse experimental settings. This tool provides an unprecedented opportunity to map viral spread and identify infected cell populations in complex tissues, such as the brain.

In this study, we tested tGFP-TBEV in cell cultures and an *ex vivo* model based on rat organotypic cultures of cerebellar slices [33]. We then used the tGFP-TBEV to investigate the spatial distribution and cellular tropism of TBEV in a mouse model. Using FDISCO, a method for tissue clearing [34], combined with light-sheet fluorescence microscopy, we mapped infected loci in the whole mouse brain after intracranial infection. Our analysis revealed distinct patterns of viral spread, with an emphasis on infection in regions associated with neurogenesis and olfactory processing. These findings provide new insights into the neuropathogenesis of TBEV, highlighting critical neuroanatomical pathways involved in viral dissemination and replication.

The development of tGFP-TBEV and its application in this study is marked progress in the tools available for TBEV research. By enabling high-resolution 3D visualization of viral infection, this approach lays the groundwork for future studies investigating the dynamics of TBEV infection, the role of specific brain regions in pathogenesis, and potential therapeutic strategies to mitigate CNS infection.

## Materials and methods

### Ethical statement

This study was conducted in strict accordance with Czech laws and guidelines for the use of laboratory animals and the protection of animals from cruelty. All animal experiments complied with the relevant European Union guidelines for working with animals. The animal care and animal use protocols adhered to the fundamental guidelines for the proper conduct of animal experiments and related activities in academic research institutes according to the Animal Welfare Act (No. 246/1992 Coll.) in the Czech Republic. All procedures were reviewed by local ethics committees and approved by the Ministry of Agriculture of the Czech Republic (permit no. 1/2024).

Animal experiments with Wistar rats were approved by the Animal Care and Experimentation Committee of Bern, Switzerland (BE 17/2023) and conducted following Swiss National Guidelines.

### Viruses

TBEV strain Hypr, a highly pathogenic strain and member of the Western European TBEV subtype, was used as a reverse-genetics template and a control for all experiments (GenBank: MT228627.1). The virus with a low passage number was provided by the Collection of Arboviruses, Institute of Parasitology, Biology Centre of The Czech Academy of Sciences, Ceske Budejovice, Czech Republic (http://www.arboviruscollection.cz). tGFP-TBEV was produced by reverse genetics and the passage after viral rescue (P1) was used for most of the experiments.

### Cells

BHK-21 (ATTC CCL10) cells were used for viral rescue and to evaluate the growth kinetics of the virus, together with the A549 (ATTC CCL 185) and SK-N-SH (ATTC HTB-11) cell lines. A549 cells were also used for *in vitro* cytopathogenic assays. For plaque assays, we used porcine kidney stable (PS) cells from the International Cell Culture Collection, National Institute of Public Health, Prague, Czech Republic. All of the above cell lines were cultured in Dulbecco’s modified Eagle medium (DMEM) supplemented with 10% newborn calf serum for the BHK-21, A549, and SK-N-SH cells or Leibovitz (L-15) medium supplemented with 3% newborn calf serum for the PS cells, as well as 100 U/ml penicillin, 100 µg/ml streptomycin, and 1% glutamine (Sigma-Aldrich, Prague, Czech Republic). BHK-21, A549, and SK-N-SH cells were cultured at 37°C in a 5% CO_2_ atmosphere, whereas PS cells were cultured at 37°C without CO_2_ supplementation.

### Reverse-genetics system for tGFP-TBEV

#### Design

**Figure 1.**
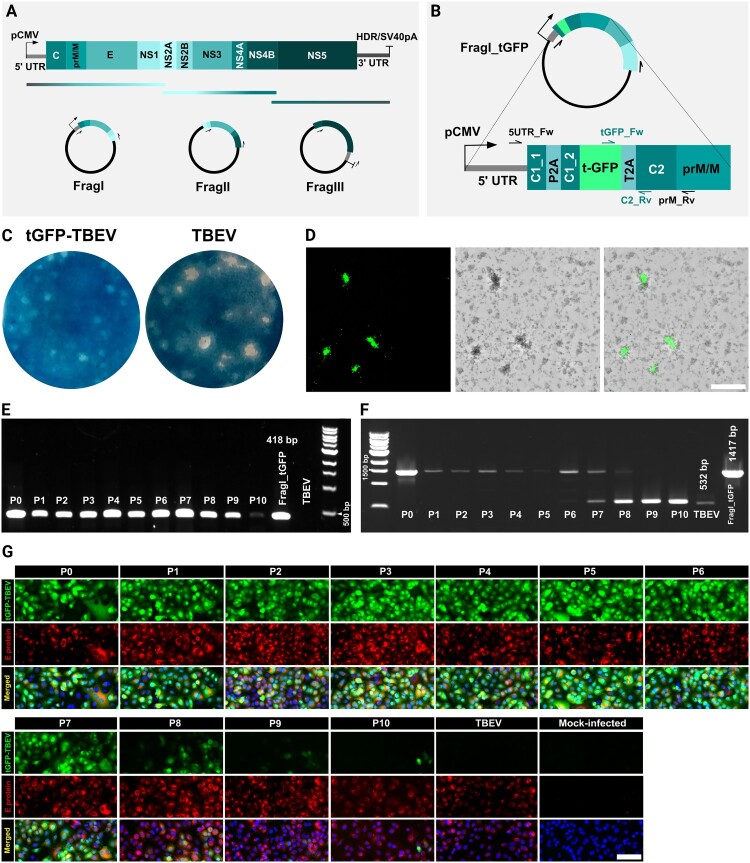
**Rescue and stability of recombinant reporter virus tGFP-TBEV.** A: Schema of a reverse-genetics system for TBEV. B: Insertion of tGFP gene into FragI. C: Difference in plaque morphology between rescued tGFP-TBEV and parental TBEV. D: Plaque assay imaged in the FITC channel and brightfield. Scale = 500 μm. E: Viral RNA from the supernatant at each passage was isolated and used for RT-PCR to amplify a partial sequence of tGFP and the C gene using primers tGFP_Fw and C2_Rv indicated in B. tGFP-TBEV was present in the samples at all 10 passages. F: The same samples were used to amplify the partial sequence of the C gene surrounding the inserted tGFP using primers 5UTR_Fw and prM_Rv indicated in B. The revertants occur at passage 7 (P7). FragI_tGFP was used as a positive control. RNA isolated from parental TBEV was used as the control. G: BHK-21 cells infected with 10 passages of tGFP-TBEV. Following infection, the cells were fixed and immunostained to detect flaviviral E protein. As controls, cells were either infected with TBEV or mock-infected. Scale = 100 μm.

The reverse-genetics system was based on the approach published previously by our laboratory [31]. This approach uses the generation of infectious, subgenomic, overlapping cDNA fragments. Briefly, the entire genome of the TBEV Hypr strain (GenBank: MT228627.1) was divided into three fragments: FragI, FragII, and FragIII. These fragments were *de novo* synthesized and cloned into pUC57 or pCC1 vectors (GenScrip, Piscataway, NJ, US). The human cytomegalovirus promoter (pCMV) was present at the 5’ end of FragI, and the combination of hepatitis delta ribozyme and simian virus 40 polyadenylation signal (HRD/SV40pV) was located at the 3’ end of FragIII (Figure 1(A)). The tGFP gene was amplified from commercial vector pTurboGFP-N (cat.# FP512, Evrogen) and assembled into FragI_tGFP using the Gibson Assembly® Cloning kit (New England Biolabs).

The tGFP gene was located within FragI downstream of the full-length 5’ UTR and the first 72 nucleotides of the C gene, which is necessary for the replication of flaviviruses, to generate FragI_tGFP. The stability of the reverse-genetics system was improved by adding P2A ribosome skipping sequence followed by another 72 nucleotides of the C gene and the full sequence of the tGFP gene. Another ribosome sequence, T2A, is present at the 3’ end of the tGFP gene (Figure 1(B)). tGFP is expressed as a translation fusion to the truncated N-terminus of the TBEV C protein [35-37].

#### tGFP-TBEV rescue

FragI_tGFP, FragII, and FragIII were amplified from the vectors and used for transfection. We used the same PCR reaction and conditions, including the same primers, as described previously [31]. PCR fragments were purified using a Wizard SV Gel and PCR Clean-Up Systems (Promega) purification kit before being used for the transfection of BHK-21 cells. The purified amplicons were mixed at an equimolar rate and the transfection reaction prepared with 2 µl of XtremeGENE^TM^ HP DNA Transfection Reagent (Roche), which was diluted in 200 µl of Opti-MEM medium (Life Technologies) and mixed with the amplicons. The reaction was incubated for 15 minutes at room temperature and then added to 80% confluent BHK-21 cells in a 24-well plate. The fluorescence was observed regularly. As the strong signal occurred on day 4, the media containing the rescued virus (P0) was collected and the titre determined by plaque assay. P0 was used for the production of P1 by sub-passaging in BHK-21 cells grown in a 75-cm^2^ flask. After 3 days, the supernatant containing tGFP-TBEV was harvested and the titre determined by plaque assay. This P1 of tGFP-TBEV was then used for most of the experiments.

### Serial passaging of tGFP-TBEV

After the tGFP-TBEV rescue, P0 was serially passaged on BHK-21 cells. Cells were plated in 24-well plates to reach 80% confluency on the day of infection. Cells were infected with P0 at a multiplicity of infection (MOI) of 0.01 to produce P1. All other passages were infected with an MOI of 0.1. The supernatant of each passage was harvested 3 days post-infection (dpi) and the titre determined by plaque assay. The serial passaging was performed 10 times (P0 – P10). Each passage was performed in triplicate, and the fluorescence of the infected cells was observed regularly.

### Plaque assay

Plaque assays were performed using PS cells as described previously [38,39]. Briefly, 10-fold dilutions of viral suspension were prepared in 24-well plates and 1 × 10^5^ cells added directly to the viral suspension in each well. After incubation at 37°C for 4 hours, the cells were overlaid with 1.5% carboxymethylcellulose in L-15 medium. After 5 days of incubation, the infected plates were washed with phosphate-buffered saline (PBS) and the cell monolayers stained with naphthalene black. The virus titre was expressed as plaque-forming units (PFU) per millilitre. A similar approach was used to determinate fluorescent focus unit (FFU)/plaque forming unit (PFU) ratio (Supplementary Fig. 1). After 5 days of incubation, the infected plates were imaged using FITC channel and brightfield to quantify the number of FFU and PFU using ImageXpress Pico Automated Cell Imaging System (Molecular Devices).

### Viral RNA isolation and RT–PCR

Viral RNA was isolated from the culture supernatant infected with the serial passages of tGFP-TBEV (P0-P10) or mouse brain tissue homogenate. For both, we used the QIAmp viral RNA mini kit (Qiagen) according to the manufacturer’s instructions. RT–PCR was performed using the QIAGEN OneStep RT–PCR Kit (Qiagen) following the manufacturer’s recommendations for a 25 µl reaction using Q-Solution and 1 µl of isolated RNA as a template. The sequences of the primers used in the reactions can be found in Supplementary Table 1. The cycling conditions depended on the amplicon length and followed the manufacturer’s instructions.

### Viral growth kinetics

#### Grow kinetics in BHK-21 cells

We compared the growth kinetics of tGFP-TBEV with the parental TBEV. The BHK-21 cells were plated in a 96-well plate at a density of 20,000 cells/well. The infection was performed using MOIs of 1 and 0.1. The supernatant from the infected cells was collected regularly every 6 hours up to 48 hours post-infection (hpi) and every 12 hours up to 96 hpi, with each time point sampled in triplicate. The samples were then assessed by plaque assay to determine the growth curves for each variant.

#### Live-cell imaging, in vitro cytopathogenic assay, and imaging of plaque formation

A similar approach was used to evaluate the fluorescent signal intensity. BHK-21, A549, or SK-N-SH cells were plated in 96-well plates (µCLEAR®, black, CELLSTAR®, Greiner BIO-ONE) at a density of 17,000 cells/well and grown overnight. Before infection, the cell nuclei were stained with Hoechst 34580 (Invitrogen) in 1:5000 dilution for 30 minutes at 37°C in a 5% CO_2_ atmosphere. After staining, the medium was replaced with FluoroBrite^TM^ DMEM (ThermoFisher Scientific) containing the virus diluted to an MOI of 0.1. The plates were then incubated and analyzed in the ImageXpress Pico Automated Cell Imaging System (Molecular Devices) using the environmental control with the following setup: 37°C, 5% CO_2_, 95% humidity.

For the *in vitro* cytopathogenicity assay, we used the same conditions as described above but with the addition of the Sytox^TM^ Deep Red Nucleic Acid Stain (ThermoFisher Scientific) diluted to 0.5 µM in the FluoroBrite^TM^ DMEM prior to virus inoculation.

For imaging of formation of fluorescent foci (Supplementary Movie S1), BHK-21 cells were seeded as mentioned above. After Hoechst 34580 nuclei staining, 100 PFU/well of tGFP-TBEV was added in FluoroBrite^TM^ DMEM media and the virus diluted in a 2-fold manner. After incubating for 90 minutes at 37°C, the cells were overlaid with 1.5% carboxymethylcellulose. The plate was incubated for 96 hours in an ImageXpress Pico environmental control under the same conditions as for live-cell imaging and imaged every 3 hours.

## Mouse infections

All mice used for the experiments were 6-week-old female BALB/c mice (Envigo). The mice were housed in standard cages in a BSL-3 facility (max. 6 mice/cage) with water and food provided ad libitum. Mice were infected with a lethal dose of tGFP-TBEV or parental TBEV (8 × 10^4^ PFU/mouse) via the intracranial route (cerebral cortex or midbrain) or intraperitoneal route. Some animals served as uninfected controls. During the 7-day experimental period, mice were monitored for health status, clinical signs of disease, and survival. After the symptoms appeared, one group of animals infected with tGFP-TBEV via intracranial route and control animals were perfused with ice-cold PBS followed by 4% w/v paraformaldehyde fixation. The brains of the perfused mice were dissected and used for tissue clearing. The rest of the animals were anaesthetized, blood samples collected, and the mice humanely killed by cervical dislocation. The brains were dissected and homogenates of the whole brain tissue prepared. Some brains were fixed in 4% w/v paraformaldehyde and tissue slices prepared. Mouse brains were homogenized to produce a 30% homogenate in culture media. In the group of intraperitoneally infected animals, blood was collected prior the euthanasia at the end of the experiment (28 days post-infection). Sera were separated and stored until ELISA analysis.

### Immunostaining

#### Mouse brain sections

The dissected mouse brains were fixed overnight in 4% w/v paraformaldehyde at 4°C. Fixed brains were washed in PBS and incubated overnight in 30% w/v sucrose solution. Next, the tissue was embedded in OCT media (6478.1, Cell Path) and immediately frozen on dry ice until cryosectioning. The slices were prepared at a thickness of 10 µm using a rotatory microtome cryostat (CM1860UV, Leica). Before staining, the tissue was blocked and permeabilized in 10% v/v goat serum, 0.2% v/v Triton X-100, and 1% w/v bovine serum albumin in PBS for 1 hour at room temperature. The antibodies listed in Supplementary Table 2 were used at the indicated dilutions together with the appropriate secondary Alexa Fluor^TM^ 647 antibody. Antibodies were diluted in 2% v/v goat serum and 0.5% v/v Triton X-100 in PBS and incubated with the samples, overnight at 4°C for the primary antibody and 1 hour at room temperature in the dark for the secondary antibody. The nuclei were counterstained with 1 µg/ml 4′,6-diamidino-2-phenylindole (DAPI; Sigma-Aldrich). The immunostained tissue slices were mounted with AntiFade mounting medium (MedChemExpress) and stored at 4°C until imaging. Fluorescence microscopy was performed using the ImageXpress Pico Automated Cell Imaging System (Molecular Devices, USA). The images were processed by ImageJ/Fiji software (version 1.54d). Co-localization analysis between tGFP and other signals was performed using the JACoP plugin in ImageJ/Fiji [40].

#### Cell monolayer

The cells were fixed for 15 minutes with chilled 4% paraformaldehyde at room temperature, permeabilized with 0.1 Triton X-100 in PBS for 10 minutes, then washed twice with PBS, and blocked with 10% foetal bovine serum in PBS for 30 minutes at 37°C. Prior to staining, the plate was washed with wash solution (PBS with 0.05% Tween 20). The primary antibody was prepared at the dilution indicated in Supplementary Table 2 and the plate incubated at 37°C for 1 hour. After washing three times with wash solution, the cells were labelled with the secondary Alexa Fluor^TM^ 647 antibody at the dilution listed in Supplementary Table 2 and then incubated at 37°C for 1 hour. The cells were counterstained with 1 µg/ml DAPI to visualize the cell nuclei. Images were acquired using the ImageXpress Pico Automated Cell Imaging System (Molecular Devices, USA). The images were processed by ImageJ/Fiji software (version 1.54d).

### Measurement of tGFP-TBEV intensity in mouse brain tissue homogenate

The mouse brain tissue homogenates were diluted in DMEM to obtain 10% solutions and centrifuged twice for 3 minutes at 13,000 × g to obtain a clear solution. These solutions were diluted 100 × in PBS and the fluorescence intensity measured by a Microplate Reader Synergy H1 (BioTek) with the following parameters: plate type: 96 WELL PLATE, excitation: 479 nm, emission: 520 nm, optics: bottom, gain: 100, light source: Xenon Flash, lamp energy: High, read speed: Normal, delay: 100 msec, temperature: 37°C.

### ELISA

The presence of specific anti-TBEV antibodies in mouse sera was assessed using ELISA IMMUNOZYM FSME (TBEV) IgG All Species Kit (Progen, Cat. No. 7701075). The ELISA kit was used according to manufacturer’s instructions. Samples with antibody concentrations below 63 VIEU/ml were considered negative.

### Whole mouse-brain clearing and light-sheet microscopy

Dissected mouse brains (n = 4) were cleared using the organic solvent-based clearing technique FDISCO as described previously [34]. First, the brains were fixed in 4% w/v paraformaldehyde overnight at 4°C and washed in ice-cold PBS. Next, the brains were incubated at 4°C in a rotated Falcon tube for 12 hours in a 50% v/v solution of tetrahydrofuran THF (Sigma) and water (pH 9). The next 12 hours of incubation was in 80% v/v THF followed by 100% THF. After dehydration, the mouse brains were transferred to dibenzyl ether (DBE) for refractive index matching. Brains were incubated with DBE for 3 hours at 4°C before being submerged in ethyl cinnamate (ECi) and observed under a light-sheet microscope (ZEISS Lightsheet 7) with 5× objective. Individual brains were held on a vertical sample holder using superglue and imaged while submerged in a sample chamber filled with ECi. The brains were imaged in sagittal orientation with only half of the brain closer to the detection objective being imaged. Image processing was achieved by Zen Blue (ZEISS). Videos from the light-sheet microscope were prepared in Imaris v10.0.0. (Oxford Instruments). Raw data from the light-sheet microscopy can be obtained from the authors upon request.

### Production and maintenance of organotypic cerebellar slices

Organotypic brain slice culture (BSC) medium was prepared by adding 1% GlutaMAX (Thermofisher, 35050-038) and 1% antibiotic-antimycotic (Thermofisher, 15240-062) to Neurobasal medium (Thermofisher, 10888-022). The dissection medium was produced by adding 6 mg/ml glucose (Thermofisher, A2494001) and 1% antibiotic-antimycotic to Hanks’ Balanced Salt Solution (HBSS) (Thermofisher, 14175-095).

Ten-day-old Wistar rats (from the Central Animal Facility of the University of Bern, Switzerland, or from Charles River, Germany) were euthanized by intraperitoneal injection of pentobarbital (Escornarkon, Streuli AG, 150 mg/kg body weight). The head was sprayed with 70% ethanol, cut off, and the brain isolated and directly immersed in ice-cold dissection medium.

The cerebellum was isolated from the rest of the brain using a scalpel and cut into two along the sagittal plane. The two cerebellar hemispheres were glued onto the vibratome cutting plate and cut in parallel (Leica VT 1000 S vibratome) with the speed set to 0.40 mm/s and the slice thickness to 325 μm. The slices were transferred onto the membrane of Transwell inserts (Sigma Aldrich, CLS3450, 3 slices per insert) kept on top of ice-cold dissection medium. After all slices were cut, the inserts were transferred to a 6-well plate containing pre-equilibrated BSC medium freshly supplemented with B27 supplement (Thermofisher, 17504-044) and the plates incubated at 37°C in a 5% CO_2_ atmosphere. The BSC medium supplemented with B27 was replaced every 2–3 days.

### Infection and immunohistochemistry of organotypic cerebellar slices

After 5 days in culture, organotypic cerebellar slices (OCSs) were infected. For this purpose, the inoculum was prepared by adding viral stock to the OCS medium (5.0 × 10^4^ PFU/ml) in a final volume of 1 ml/well. Old medium was removed from the wells and 0.8 ml inoculum added at the bottom of the well; the remaining 200 μl were evenly distributed on top of the slices. The plates were incubated at 37°C and 5% CO_2_ for 1 hour. Following the incubation period, the inoculum was removed from the top and bottom of the slices. The wells were washed once with 2 ml PBS. To the bottom of each Transwell insert, we added 1 ml of BSC medium supplemented with B27 (20 μl/ml). The plates were incubated for 4 days without further changes to the medium. Subsequently, the slices were fixed directly on the inserts with 4% paraformaldehyde for 90 minutes and then kept in PBS for up to 3 days.

Fixed samples were initially incubated in blocking buffer (10% FBS, 2% DMSO, 2% Triton X-100 in PBS) for 2 hours at room temperature. Subsequently, they were incubated with primary antibodies (Supplementary Table 2) in blocking buffer for 48 hours on a shaker at 4°C. Following this, they were washed three times with PBS for 20 minutes each, followed by a 12-hour incubation in PBS at 4°C. They were then incubated in blocking buffer for 2 hours. Secondary antibodies (Supplementary Table 2) in blocking buffer were added and the samples incubated on a shaker at 4°C for another 24 hours. At the end of the incubation, the samples were washed with at least five changes of PBS over 4 hours at 4°C. Thereafter, the samples were incubated in PBS overnight at 4°C and mounted on microscope slides with Fluoroshield (Sigma Aldrich, F6182).

Images were taken with a confocal microscope (Leica digital light sheet TCS SP8 DLS) using a 20× water immersion objective. The confocal images were deconvoluted using the Huygens Remote Manager. Images were finally elaborated using ImageJ/Fiji software. Co-localization analysis between tGFP and signal for E protein was performed using the JACoP plugin in ImageJ/Fiji [40].

### Statistical analysis

All data are expressed as means and standard deviations. Differences between groups were evaluated using the Mann–Whitney U test. All tests were performed in GraphPad Prism 7 (version 7.04; GraphPad Software, Inc., La Jolla, CA, USA). Differences with p < 0.05 were considered significant.

## Results

### Rescue of recombinant reporter virus tGFP-TBEV and stability analysis during serial passages

To generate the recombinant tGFP-TBEV, we utilized a reverse-genetics system previously established for TBEV rescue. This system is based on infectious, subgenomic, overlapping DNA fragments covering the entire viral genome [41,42] and was previously used for the creation of mCherry-TBEV [31]. However, in the tGFP-TBEV construct, the mCherry gene is replaced with the tGFP gene, and a duplicated C1_1 fragment (72 nt) of the capsid (C) gene is inserted immediately upstream of the tGFP gene (Figure 1(A, B)). Our preliminary experiments indicated that this duplication significantly enhances the stability of the insertion up to 7–10 passages (see below). In contrast, mCherry-TBEV without the duplication was stable in cell culture for fewer than four passages [31].

Three days after transfection, viral titres in the supernatant were measured by a plaque assay on PS cells, yielding a titre of 8.2 × 10^7^ PFU/ml at passage zero (P0). Co-localization of tGFP fluorescence with flaviviral E protein staining confirmed the successful rescue of tGFP-TBEV (Figure 1(G)). The supernatant was then used to infect fresh BHK-21 cells, producing a P1 virus with a titre of 4.2 × 10^6^ PFU/ml. The tGFP-TBEV plaques were smaller than the plaques of the parental TBEV strain (Figure 1(C)). Fluorescent foci in plaque assays enabled titration as fluorescent focus units (FFU) per millilitre 5 dpi (Figure 1(D)). Fluorescent foci formation is visualized in Supplementary Movie S1.

To evaluate the stability of tGFP-TBEV, we performed 10 serial passages in BHK-21 cells. The virus had significantly improved stability compared to mCherry-TBEV, which lost the reporter gene after four passages [31]. RT–PCR analysis of samples collected during serial passages confirmed the presence of tGFP-TBEV for at least 10 passages (Figure 1(E, F)). The viral titre in serial passages remained between 1.4 × 10^7^ and 2.4 × 10^8^ PFU/ml in all passages (Supplementary Fig. 1A). The tGFP-positive cells were detected across all 10 passages, but the proportion of fluorescent cells declined over time, with a marked fluorescent decrease observed in passage 8 (Figure 1(G)).

Despite the enhanced stability of tGFP-TBEV, homologous recombination led to the emergence of revertant variants. This decline in fluorescence correlates with RT–PCR analysis, which confirmed a presence of revertants beginning passage 7 (Figure 1(F)). A similar trend was observed in the fluorescent focus units relative to plaque-forming units (FFU/PFU) starting form passage 7 and more prominently at passage 8 (Supplementary Fig. 1B and C). This finding is consistent with the emergence of revertants and the loss of tGFP expression described above. Sequencing of late-passage samples revealed recombination events between the ribosome-skipping sites P2A and T2A (Supplementary Fig. 2). These findings demonstrate that adding the C1 fragment upstream of tGFP significantly improves the stability of the reporter virus while allowing the detection of recombination events at later passages.

### Growth and cytopathogenicity of tGFP-TBEV

**Figure 2.**
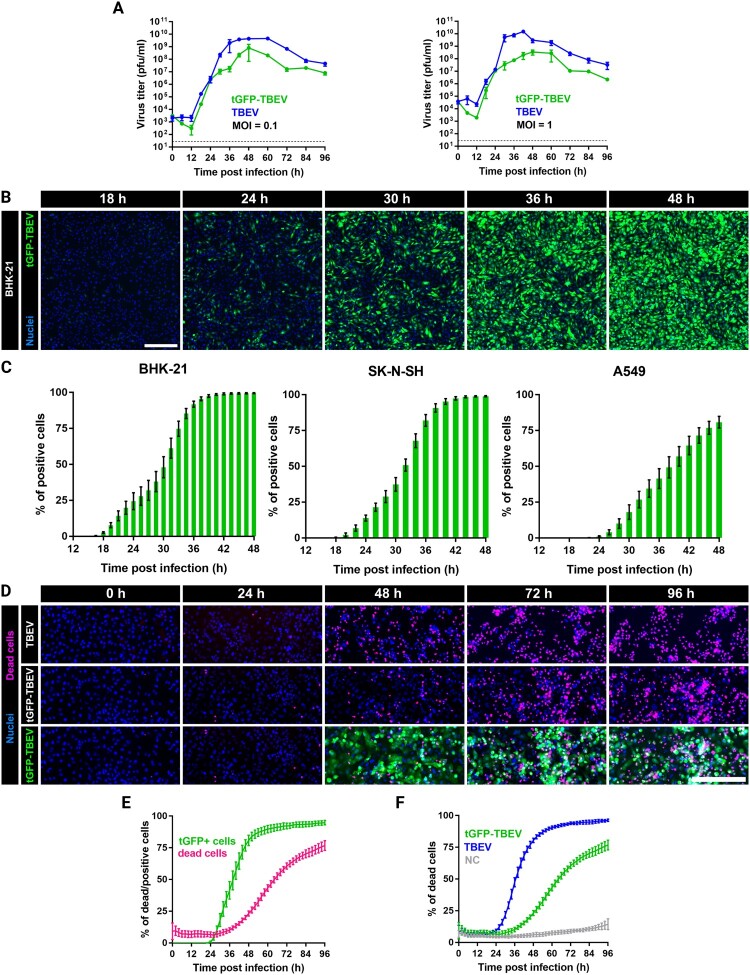
**Characterization of the growth properties and cytopathogenicity of tGFP-TBEV.** A: Growth kinetics of tGFP-TBEV infection compared with parental TBEV characterized in BHK-21 cells at MOI = 1 and MOI = 0.1, (n = 3). B: Real-time visualization of infection in live BHK-21 cells. Cells were infected with tGFP-TBEV (MOI = 0.1) and imaged every 6 h. Scale bar = 300 μm. C: Percentage of tGFP-positive cells detected during infection among BHK-21 (n = 2 × 6), SK-N-SH (n = 2 × 12), and A549 cells (n = 2 × 8). D: *In vitro* cytopathogenicity assay visualized in real-time in live A549 cells. Scale bar = 300 μm. E: *In vitro* cytopathogenicity assay showing the tGFP signal in tGFP-TBEV-infected samples compared to the percentage of dead cells, (n = 2 × 8). F: *In vitro* cytopathogenicity assay in live A549 cells comparing the virulence of both tGFP-TBEV and parental TBEV to mock-infected cells as the percentage of dead cells over time, (n = 2 × 8).

The growth properties of tGFP-TBEV were evaluated in BHK-21 cells and compared with the parental TBEV strain. Cells were infected at MOIs of 0.1 and 1. At both MOIs, tGFP-TBEV exhibited peak infection 48 hpi, with titres of ∼10^8^ PFU/ml. At both MOIs, tGFP-TBEV had reduced peak titres compared to parental TBEV, but the overall growth kinetics were comparable (Figure 2(A)).

To compare viral replication using fluorescence signal dynamics, live-cell imaging was performed for infections at an MOI of 0.1. Fluorescence data confirmed the infection peak, with the percentage of infected cells reaching the maximum before 48 hpi (Figure 2(B and C), Supplementary Movie S2). The first GFP-positive cells were detectable as early as 18 hpi, correlating with a titre of 2.6 × 10^4^ PFU/ml. Infections performed in different cell lines, including human neuroblastoma SK-N-SH cells and A549 lung epithelial cells, demonstrated that tGFP-TBEV can infect multiple cell types, though the percentage of GFP-positive cells and the viral growth kinetics varied between cell lines.

To assess the virulence of tGFP-TBEV, we performed an *in vitro* assay combining live-cell imaging with Sytox™ Deep Red Nucleic Acid Stain (ThermoFisher Scientific) to detect dead cells. The virulence of tGFP-TBEV was determined by measuring the percentage of infected (tGFP-positive) and dead cells over time. A549 cells were selected for this assay because their slower infection kinetics provide an extended timeframe that is more conducive to detailed analysis and observation of viral replication dynamics.

tGFP-TBEV infection resulted in 50% of cells being GFP-positive by 38 hpi, with cell death lagging by approximately 24 h (Figure 2(E)). This delay increased as the infection progressed. Compared to the parental TBEV strain, tGFP-TBEV exhibited lower cytopathogenicity. At 48 hpi, the percentage of dead cells was significantly lower in tGFP-TBEV-infected cultures than in parental TBEV cultures (23% vs. ∼80%; Figure 2(D and F)).

These results indicate that, though tGFP-TBEV retains robust infectivity and replicative capacity, it demonstrates reduced cytopathogenicity compared to the parental TBEV strain. This slight attenuation makes tGFP-TBEV a valuable tool for studying viral infection dynamics and pathogenesis *in vitro*, offering an extended timeframe for analyses by reducing the extensive early cell deaths frequently seen after infection with wild-type TBEV.

### tGFP-TBEV infection in rat organotypic cerebellar slices ex vivo

**Figure 3.**
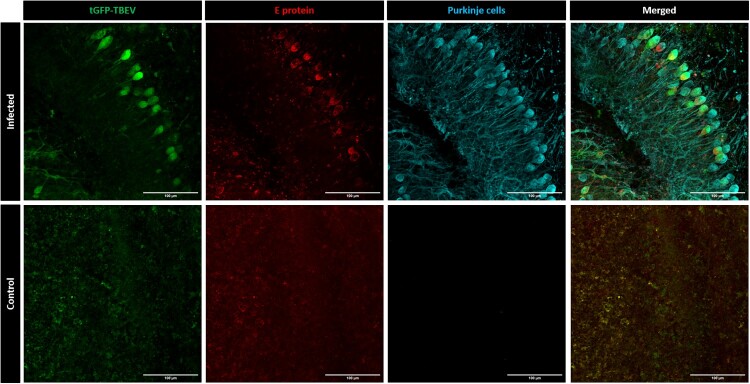
tGFP-TBEV infection of rat organotypic cerebellar slices (OCSs). OCSs from Wistar rats infected with tGFP-TBEV were compared with mock-infected samples. The 4G2 antibody was used to visualize TBEV envelope antigen. Calbindin antibody was used to specifically stain Purkinje cells. Scale = 100 μm.

To investigate tGFP-TBEV infection in a more complex model, we cultured rat OCSs [33] *ex vivo* and infected them with tGFP-TBEV. This approach, previously used to study infection with the highly pathogenic TBEV strain Hypr [33], allowed for the exploration and localization of TBEV infection within the brain tissue and facilitated the identification of cell types most susceptible to the virus. Following infection, OCSs were stained for TBEV E antigen and calbindin, a marker in Purkinje cells (Figure 3). A strong co-localization was observed between the tGFP fluorescent signal and TBEV staining, confirming the specificity of the reporter virus. Notably, staining for Purkinje cells revealed that these neurons were particularly vulnerable to TBEV infection in this *ex vivo* model, same as was described previously for TBEV strain Hypr [33]. Interestingly, while the tGFP signal was evenly distributed within the infected cell bodies, the E protein signal appeared more confined, likely localized to the cytoplasm or endoplasmic reticulum, and excluded from the nucleus. Co-localization analysis yielded a Pearson’s correlation coefficient *r* = 0.605, indicating a strong correlation between reporter expression and viral protein production. Additionally, thresholded Mander’s overlap coefficients (M1 = 0.578, tGFP signal overlapping E protein signal; M2 = 0.643, E protein signal overlapping tGFP signal) confirmed substantial overlap of high-intensity signals, underscoring the specificity of tGFP signal as a marker of productive infection.

### tGFP-TBEV is pathogenic for mice after intracerebral inoculation

**Figure 4.**
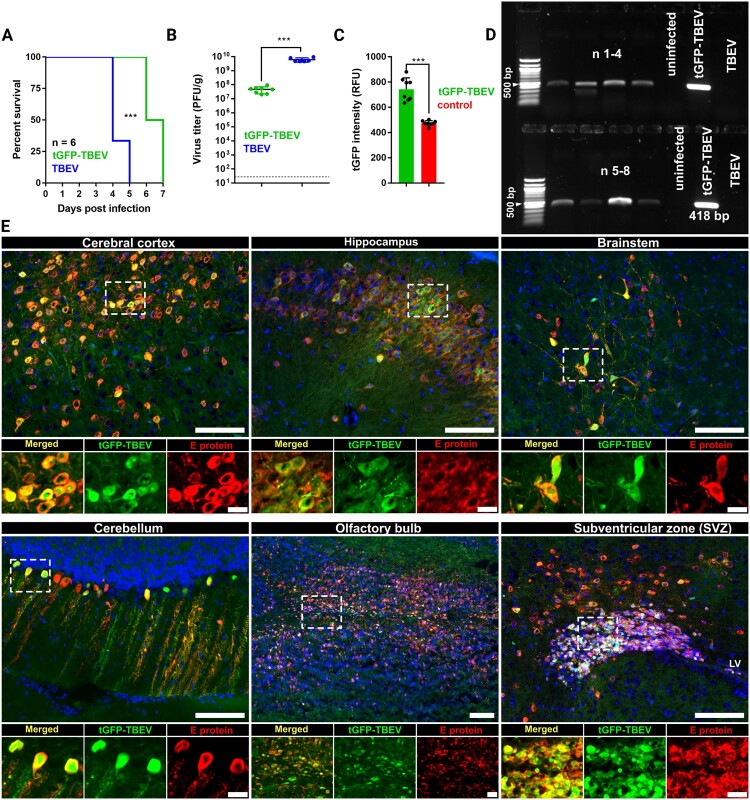
**tGFP-TBEV-infection of mice.** A: Percent survival of mice showing pathogenicity of the recombinant virus compared with parental TBEV. B: Quantification of virus in mouse brain homogenate using plaque assay, (n = 8). C: Intensity of the tGFP fluorescent signal in mouse brain homogenate compared with uninfected control brains, (n = 8). D: RT-PCR of RNA samples isolated from the mouse brains infected with tGFP-TBEV at P1, proving the presence of tGFP-TBEV in mouse brains. Uninfected brain was used as a control. RNA isolated from parental TBEV was used as the negative control. E: Fluorescent images of mouse brain tissue slices showing various infected regions. Tissue slices were immunolabelled for flaviviral E protein (red), showing co-localization with t-GFP signal (green). Scale bar = 100 μm or 20 μm. White frames indicate the zoomed in area. Representative images of immunostained brain sections are shown (n of the brains analyzed = 5).

In intraperitoneally infected animals with tGFP-TBEV, no symptoms occurred. To assess the neuropathogenicity of tGFP-TBEV, mice were inoculated intracranially (right cerebral cortex or midbrain), with a lethal dose of the recombinant virus, and survival rates were compared to mice infected with parental TBEV. All intracranially infected mice exhibited neurological symptoms, including ruffled fur, hunched posture, and/or paralysis, within 7 days post-inoculation, necessitating euthanasia in most cases. The mortality rate reached 100% in both groups. Notably, tGFP-TBEV infection resulted in delayed symptom onset and longer mean survival time compared to parental TBEV (*p* < 0.001), though it ultimately had the same fatal outcome (Figure 4(A)).

Plaque assays of brain tissue from infected mice revealed approximately 2 log_10_ PFU/g lower viral titres in tGFP-TBEV-infected mice (5.4 × 10^7^ PFU/g) compared to parental TBEV-infected mice (4.6 × 10^9^ PFU/g, *p* < 0.001), reflecting the overall lower virulence of the reporter virus compared to the parental TBEV (Figure 4(B)). Fluorescence measurements also showed a significant (*p* < 0.001) increase in the tGFP signal in brain homogenates from tGFP-TBEV-infected mice compared to uninfected controls (Figure 4(C)). Viral RNA containing the tGFP gene was detected in the brain homogenates of tGFP-TBEV-infected mice, confirming the presence of the reporter virus (Figure 4(D)).

Interestingly, during our preliminary experiments, mice inoculated intraperitoneally with tGFP-TBEV presented with no symptoms of infection, indicating limited peripheral replication and/or an effective innate immune response. At the end of the experiment, mouse sera were investigated by ELISA and all samples tested negative for specific antibodies (n = 4). This suggests that the intracerebral model is more suitable for analysing tGFP-TBEV infection in the CNS, as peripheral routes of inoculation fail to support sufficient viral amplification or dissemination. Consequently, the intracerebral model is ideal for studying brain-specific viral infection and associated pathogenicity.

### tGFP-TBEV exhibits neuronal tropism across various brain regions

The progression of tGFP-TBEV infection in mouse brains was analyzed using tissue sections immunostaining to confirm signal specifity and identify infected cell types. Interestingly, the strongest signal in the brain was detected in mice exhibiting the most severe symptoms. In contrast, animals sacrificed at the onset of clinical signs showed markedly weaker brain signals. This observation further supports a correlation between disease progression and reporter signal intensity (Supplementary Fig. 7).

The specifity of the t-GFP signal was validated by its co-localization with anti-E protein immunostaining in multiple brain regions (Figure 4(E), Supplementary Fig. 3). Co-localization analysis showed high degree of overlap between the signals in hippocampus (Supplementary Fig. 3, Pearson’s coeffiecient *r* = 0.797, thresholded Mander’s overlap coefficients M1 = 0.790 [green/red] and M2 = 0.767 [red/green]), cerebral cortex (*r* = 0.745, M1 = 0.818, M2 = 0.0.789) and subventricular zone (SVZ) (*r* = 0.666, M1 = 0.820, M2 = 0.688). Infection was also observed and confirmed via anti-E protein immunostaining in olfactory bulbs and cerebellum (Figure 4(E)).

**Figure 5.**
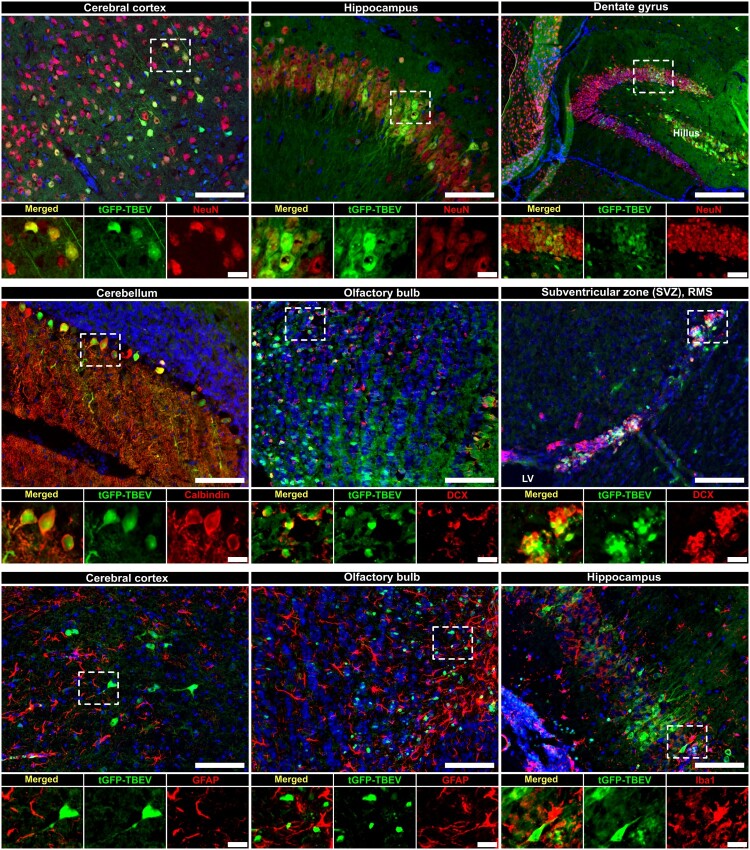
**Cell specificity in the different tGFP-TBEV infected brain regions.** Fluorescent images of mouse brain tissue sections showing various cell types within infected regions. Tissue slices were immunolabelled with anti-calbindin (red)/anti-NeuN (red)/ anti-DCX antibodies/ anti-GFAP antibodies/ anti-Iba1 antibodies, demonstrating co-localization with tGFP signal (green). Scale bar = 100 μm or 20 μm. LV = lateral ventricle. Representative images are shown (n of the brains analyzed = 5).

In addition to the SVZ, tGFP positive cells were also detected in other areas of neurogenesis, such the lateral ventricles and the subgranular zone (SGZ) surrounding the dentate gyrus. As all these regions contain immature neurons, immunostaining was performed using the immature neuronal marker doublecortin (DCX). tGFP-TBEV signal co-localized strongly with the DCX marker in SVZ (Figure 5, Supplementary Fig. 5, *r* = 0.504, M1 = 0.765, M2 = 0.618). In contrast, no co-localization with NeuN (a marker of mature neurons) was observed in this region (Supplementary Fig. 4, *r* = −0.043, M1 = 0.016, M2 = 0.049), suggesting specific infection of immature neurons in SVZ.

Although tGFP signal is visible in the dentate gyrus (Figure 5), only a few cells co-localized visually with DCX, and quantitative co-localization analysis indicated low overlap (Supplementary Fig. 5, *r* = 0.177, M1 = 0.073, M2 = 0.597) suggesting that infection in this area primarily affected mature neurons nearby rather than immature neurons. In lateral ventricle, the signal showed higher co-localization compared to SGZ (Supplementary Fig. 5, *r* = 0.454, M1 = 0.670, M2 = 0.452). Visual overlap between tGFP and DCX signal was also present in olfactory bulb and rostral migratory strain (RMS) adjacent SVZ (Figure 5).

Immunostaining further confirmed infection of pyramidal neurons in the hippocampus (Figure 4(E), Supplementary Fig. 4, *r* = 0.603, M1 = 0.796, M2 = 0.648) and cerebral cortex (*r* = 0.527, M1 = 0.851, M2 = 0.489), whereas Purkinje cells in the cerebellum were confirmed to be infected through visual overlap of tGFP signals with calbindin-positive staining (Figure 5).

tGFP-TBEV signal was also present in midbrain near the intracranial inoculation site. Notably, infection extended to the brainstem, specifically in the nuclear zone within the mesopontine tegmentum (Figure 4(E)).

Infected cells were predominately identified as neurons, as indicated by co-localization with the neuronal marker NeuN and the flaviviral E protein signal (Figure 4(E) and Figure 5, Supplementary Fig. 3 and 4). In addition, co-localization with DCX confirmed infection of immature neurons, particularly in the SVZ and lateral ventricle (Supplementary Fig. 5). No evidence of infection was observed in astrocytes or microglia (Figure 5, Supplementary Fig. 6, *r* = 0.023, M1 = 0.040, M2 = 0.030 for astrocytes in olfactory bulb, r = 0.038, M1 = 0.057, M2 = 0.061 for hippocampal astrocytes and r = 0.198, M1 = 0.147, M2 = 0.120 for microglia in cerebral cortex) highlighting a specific tropism of tGFP-TBEV for neurons in this model.

### 3D visualization of tGFP-TBEV-infected regions in whole mouse brain

To comprehensively map the spread of tGFP-TBEV infection across the entire mouse brain, we established a 3D imaging protocol utilizing light-sheet fluorescence microscopy, which enables high-resolution visualization of fluorescent signals in large, optically cleared biological samples. Following the appearance of severe neurological signs in infected mice, the brains were harvested, fixed, and subjected to a tissue-clearing process to render them optically transparent. This clearing procedure facilitated the unobstructed passage of light through the tissue, allowing us to capture fine details of the fluorescent signals originating from tGFP expression within infected cells.

**Figure 6.**
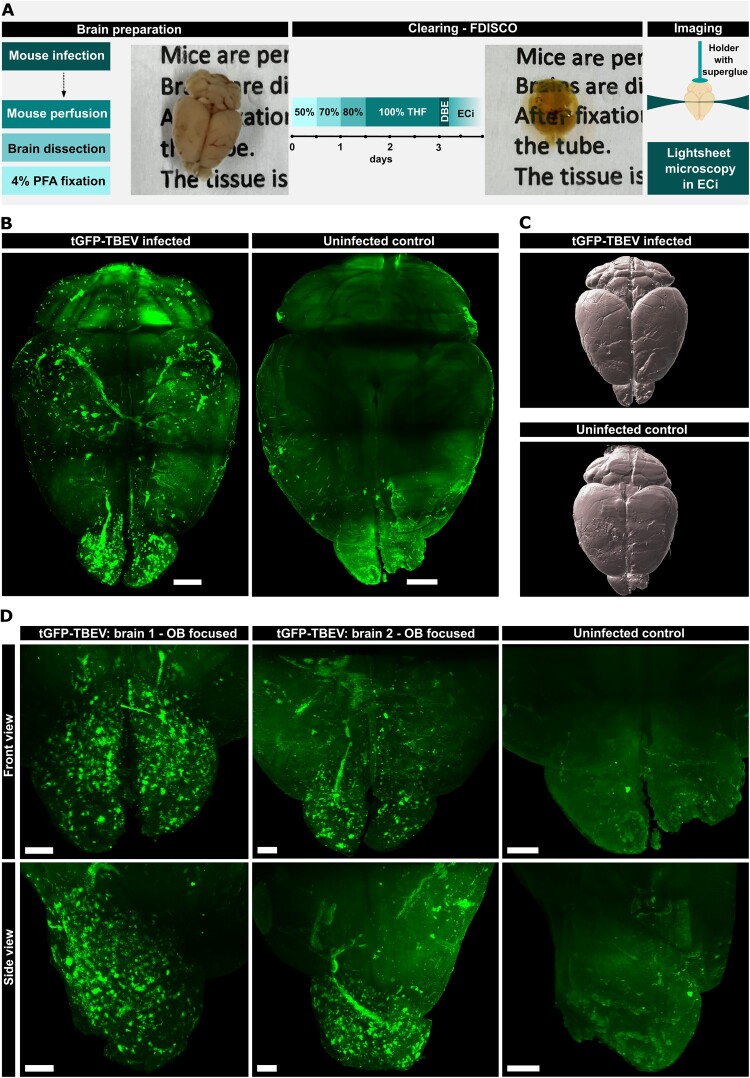
**Visualization of infected loci in olfactory bulbs (OBs) and whole mouse brain**. A: Schema of the workflow prior to light-sheet imaging with images of the brain before and after clearing. B: Visualization of tGFP-TBEV-infected whole brain compared with uninfected control. Scale bar = 1000 µm. C: Visualization of the surface of infected and control brains. D: 3D visualization of tGFP-TBEV-infected olfactory bubs imaged by light-sheet microscopy compared with control brain, visualized from the front and side. Images of two different TBEV-infected and one mock-infected brains are shown (from n = 3 TBEV-infected and n = 1 mock-infected). Scale bar = 500 µm.

**Figure 7.**
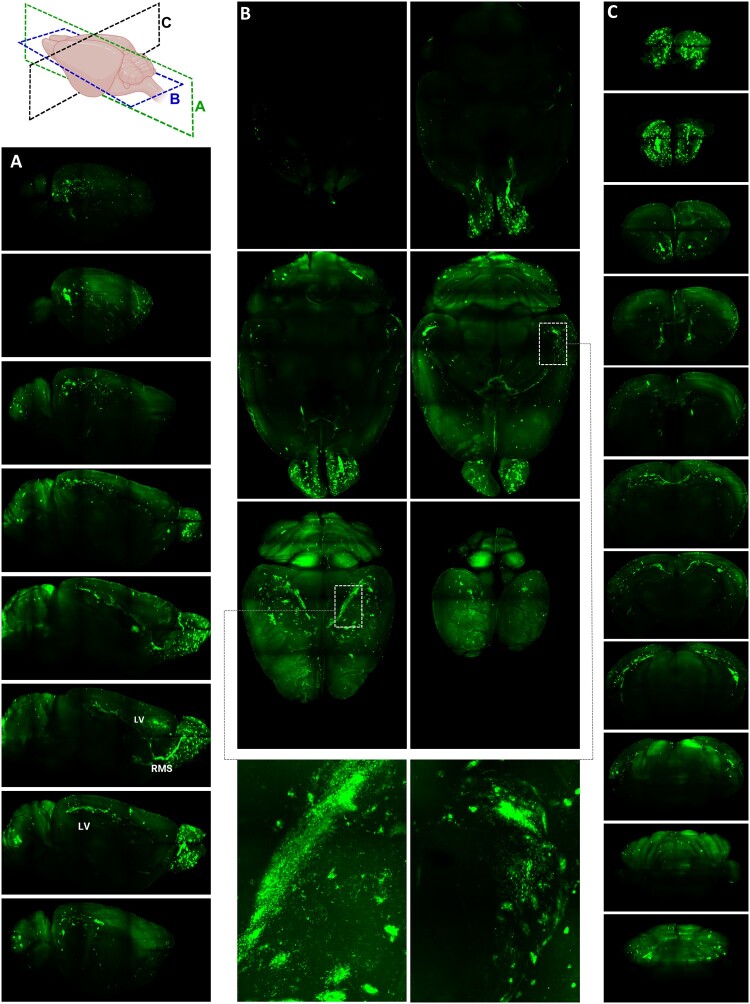
**3D visualization of infected loci in whole mouse brain sections using light-sheet microscopy.** Sections of cleared mouse brain show the internal localization of signal in different regions. A: Sagittal sections. B: Horizontal sections with zoomed in views of infected neurons in the hippocampal region. C: Coronal sections. LV = lateral ventricle, RMS = rostral migratory strain.

Our analysis revealed that infection was most pronounced in specific brain regions. Significant tGFP signals were observed in the areas surrounding the lateral ventricles, cerebellum, and hippocampus. In addition, a striking accumulation of tGFP fluorescence was detected along the rostral migratory streams (RMS), implicating that this pathway is a conduit for viral spread and infection of immature neurons. Infected cells were detected in both the SGZ and SVZ. Infection was also present in or near the corpus callosum (Figures 6 and 7, Supplementary Movie S3).

In the olfactory bulbs, intense fluorescence was observed consistently across all analyzed brains, suggesting a progression of infection from the ventricular regions to the olfactory system via the RMS after intracranial inoculation into the cerebral cortex or midbrain. This pattern underscores the potential role of ventricular regions as reservoirs or hubs for TBEV replication and the dissemination of infection to interconnected neural circuits (Figures 6 and 7, Supplementary Movie S3). Interestingly, the infection was largely confined to the ventricular-olfactory axis and did not extend significantly into other distant brain regions.

## Discussion

Development of the reporter virus tGFP-TBEV has opened up new avenues for studying TBEV infection and TBE pathogenesis in the CNS. Using this approach, we aimed to address several limitations that are usually associated with traditional, or even advanced, methods of studying TBEV infection [10]. Previous studies on reporter orthoflaviviruses have utilized various fluorescent proteins, including GFP, eGFP, mCherry, mAmetrine, miRFP703, LSSmKate2, mCardinal, and mNeptune2, as well as bioluminescent proteins, such as NanoLuc luciferase, HiBiT luciferase, Renilla luciferase, and firefly luciferase [24,26-30,43]. In our study, we used the tGFP protein, a rapidly maturing, highly soluble, and enhanced variant of a fluorescent protein derived from *Copepoda* sp., which provides a bright, long-lasting green fluorescent signal [44]. The position of tGFP insertion into the viral genome was similar to previous studies involving TBEV, as well as some other orthoflaviviruses, such as Japanese encephalitis or dengue viruses [26,27,29-31]. However, incorporating the reporter gene into the viral genome introduces selective pressure, often leading to the spontaneous deletion of non-essential regions of the inserted gene to optimize viral replication [24]. This feature represents a critical challenge in the field, where reporter orthoflaviviruses often lose their genetic insertions over time, limiting their experimental applicability [27,31]. Previously, the reporter gene being flanked with ribosome-skipping 2A sequences was shown to lead to increased stability of the reporter virus [26]. In addition, we duplicated the C1_1 fragment of the C gene before the tGFP gene, which seems to be instrumental in improving the stability of the insert, as tGFP-TBEV exhibited considerably enhanced genetic stability compared to the previously developed mCherry-TBEV in which this fragment was not duplicated [31]. Similar modification can be done also in mCherry-TBEV to increase stability of the reporter.

Our experiments confirmed that tGFP-TBEV retains the ability to replicate efficiently in cell culture, achieving growth kinetics comparable to the parental TBEV. However, it exhibited slightly lower peak titres and formed smaller plaques. These findings align with previous reports indicating that the insertion of reporter genes into orthoflavivirus genomes can attenuate viral replication, often manifesting as slower growth rates and reduced plaque sizes compared to the wild-type virus [24,27,29,31,45]. Reporter orthoflaviviruses typically demonstrate varying degrees of attenuation *in vivo* [16,46], and tGFP-TBEV is no exception. Following peripheral inoculation, all mice survived, indicating limited systemic replication of the reporter virus. This limits the ability to investigate TBEV infection in peripheral organs using immunocompetent such as BALB/c mice. Usage of immunocompetent mice such as *Ifnar ^–/–^*, could help to overcome this limitation.

In contrast, intracranial inoculation resulted in 100% mortality. However, the onset of symptoms of neuroinfection was delayed and the mean survival time extended compared to the parental TBEV. This attenuation highlights reduced pathogenicity *in vivo*, which can be paradoxically advantageous for some experimental applications by providing a more controlled model for studying viral dynamics within the CNS.

Since intracranial infection with the reporter virus remains the only available method to visualize infection in immunocompetent mice such as BALB/c, it may not fully recapitulate the natural course of TBEV infection. This approach limits the ability to study systemic viral dissemination and pathology in peripheral organs.

tGFP-TBEV demonstrates significant potential as a versatile tool for various aspects of TBEV research. It can facilitate quantitative measurement of viral replication, enable precise tracking of individual infected cells, and support high-throughput screening of potential antiviral compounds, as well as virus-neutralization assays [31]. The use of TurboGFP as a reporter allows for straightforward visualization of infection through fluorescence microscopy or fluorescence readers, eliminating the need to evaluate classical cytopathic effects or the use of more labour-intensive methods, such as plaque assays or antigen-based immunoassays [24,28]. This simplified and efficient approach enhances the practicality of tGFP-TBEV for both fundamental research and translational applications.

In addition to its applications in *in vitro* methodologies, tGFP-TBEV was demonstrated to be a valuable tool for studying TBE pathogenesis in both *ex vivo* and *in vivo* experimental systems. Its fluorescent properties enable analysis of viral infection dynamics within these complex biological environments, facilitating the exploration of viral tropism, viral spread, and interactions with host tissues. We employed OCSs derived from suckling Wistar rats and cultured *ex vivo* to investigate tGFP-TBEV infection and tropism. OCSs have been established as a reliable model for studying LGTV and TBEV infection and testing antivirals, as they retain the natural architecture of the cerebellum [13,33]. Utilizing this model, we identified Purkinje cells as the cell type most susceptible to tGFP-TBEV infection within the rat cerebellum. This finding is consistent with observations made using wild-type TBEV in OCSs [33] and aligns with the tropism described in human fatal TBE cases [47].

However, *in vitro* or *ex vivo* techniques do not provide insights into brain viral infection and pathogenesis at the whole-organ level and do not account for the presence of different anatomical and immunological barriers. To address this limitation, we intracranially infected mice with tGFP-TBEV and processed the infected brains using tissue-clearing techniques to render even the deepest structures optically accessible and, thus, suitable for whole-brain 3D imaging. This approach allowed us to visualize the spatial distribution and dynamics of viral infection across the entire brain. A similar approach was employed to study LGTV-infected mouse brains, but it required labour-intensive immunofluorescent staining of viral antigens across the entire brain [10]. In contrast, our use of tGFP-TBEV eliminates the need for such staining, leveraging the inherent fluorescence of the reporter protein for direct visualization. Significant tGFP signals were observed in areas surrounding the lateral ventricles and hippocampus, with a particularly high accumulation of tGFP fluorescence along the RMS. Overall, tGFP-TBEV infection in the brain was significantly more pronounced than the Langat virus infection previously reported in the brains of wild-type mice. In the latter case, the infection was mainly localized to the cerebral cortex, with only weak signals observed in the olfactory bulb and no detectable infection present in the lateral ventricles or the fourth ventricle [10]. In contrast, infection of brains from *Ifnar* ^–/–^ mice with Langat virus revealed a weak infection throughout the cerebral cortex, accompanied by pronounced infection in the meninges, third ventricle, fourth ventricle, and inner wall of the lateral ventricles. This infection extended into the anterior cerebrum and olfactory bulbs, exhibiting a pattern closely resembling the RMS [10,48]. Notably, this pattern closely resembles our observations in wild-type brains infected with tGFP-TBEV, suggesting that the pattern of brain infection with Langat virus is more comparable to TBEV infection only in *Ifnar*^*–/–*^ mice [10]. However, it should be noted that intracranial infection may not fully recapitulate the course of natural infection, which represents one of the limitations of our study. In particular, although injection into the right cerebral cortex is a commonly used and standardized approach, it does not necessarily reflect the initial sites of viral entry or spread in the brain following natural infection.

Infection in the olfactory bulbs and RMS is of particular interest [48]. The RMS is a critical pathway for the migration of newly formed neurons from the SVZ in the lateral ventricles to the olfactory bulbs. The SVZ contains neural stem cells, which act as precursors to new neurons. Together with the SGZ around the dentate gyrus, the SVZ represents a key site of neurogenesis in the postnatal mouse brain. Both of these neurogenic niches are highly vascularized and situated in close proximity to cerebrospinal fluid, providing an environment conducive to neural development and regeneration [49,50]. Thus, TBEV may exhibit tropism towards mouse brain regions containing immature neurons, which has been observed in brains infected with Langat virus [10]. Further investigation into TBEV infection of immature neurons could offer valuable insights into the viral infection process within the mouse brain. Similar to Zika virus infection, the expression of key factors facilitating viral entry into cells may be significantly downregulated in mature neurons compared to immature neurons and neuroblasts [51,52].

In conclusion, the development of tGFP-TBEV represents marked progress in the study of TBE pathogenesis and infection dynamics. By combining genetic stability, a robust fluorescent signal, and efficient replication, tGFP-TBEV addresses the limitations of the earlier reporter virus mCherry-TBEV, enabling more precise and accessible studies both *in vitro* and *in vivo*. The ability to visualize infection at the cellular and whole-organ levels facilitates detailed investigations into viral tropism, replication, and interaction with host tissues, paving the way for a deeper understanding of TBEV biology. Future studies leveraging tGFP-TBEV, particularly in immature neuronal populations and neurogenic regions, could shed light on the mechanisms of TBEV neurotropism and TBE pathogenesis.

## Supplementary Material

Supplemental Material
