# Supplementary material for: Three-dimensional mapping of tick-borne encephalitis virus distribution in the mouse brain using a newly engineered TurboGFP reporter virus
Source: Emerg Microbes Infect. 2025 Jul 31;14(1):2542246. doi: 10.1080/22221751.2025.2542246 (PMC12442459; doi:10.1080/22221751.2025.2542246)
Supplement: Supplemental Material [file TEMI_A_2542246_SM9695.docx]

**Supplementary data**

**Three-dimensional mapping of tick-borne encephalitis virus distribution in the mouse brain using a newly engineered TurboGFP reporter virus**

Michaela Berankova,^1,2,3^ Simone Leoni,^4,5,6^ Jiri Holoubek,^1,2,3^ Jan Haviernik,^3#^ Jiri Salat,^1,2,3^ Denis Grandgirard,^4,5^ Stephen L. Leib^4,5^ & Daniel Ruzek^1,2,3, 🖂^

(1) Department of Experimental Biology, Faculty of Science, Masaryk University, Brno, Czech Republic.

(2) Laboratory of Arbovirology, Institute of Parasitology, Biology Centre of the Czech Academy of Sciences, Ceske Budejovice, Czech Republic.

(3) Laboratory of Emerging Viral Diseases, Veterinary Research Institute, Brno, Czech Republic.

(4) Institute for Infectious Diseases, University of Bern, Bern, Switzerland.

(5) Multidisciplinary Center for Infectious Diseases, University of Bern, Bern, Switzerland.

(6) Graduate School for Cellular and Biomedical Sciences, University of Bern, Bern, Switzerland.

#Present address: School of Biological Sciences, University of Canterbury, Christchurch 8140, New Zealand.

🖂Author for Correspondence; [ruzekd@paru.cas.cz](mailto:ruzekd@paru.cas.cz)

**Supplementary movies**

**Supplementary Movie S1: Formation of fluorescent foci.** BHK-21 cells were seeded at 17,000 cells per well and grown overnight. The next day, the cells in the first row were infected with tGFP-TBEV for 100 PFU/well. A 2-fold dilution of the viral suspension was prepared and added to the cells. Carboxymethylcellulose was added over the viral suspension to prevent the spread of the new virus particles. The 96-well plate was incubated using the environmental control with the following settings: 37 °C, 5% CO2, 95% humidity. The cell nuclei were stained with Hoechst 34580. The movie was created using ImageJ/Fiji (version 1.54d).

<https://drive.google.com/file/d/1VGBsCFKnYNZvIWXXsENRx8itB3vXd5N1/view?usp=drive_link>

**Supplementary Movie S2: Live cell imaging.** Real-time visualization of tGFP-TBEV infection in live BHK-21 cells. Cells were infected with tGFP-TBEV (MOI = 0.1) and imaged every 90 minutes using the ImageXpress Pico Automated Cell Imaging System (Molecular Devices, USA). The 96-well plate was incubated using the environmental control with the following settings: 37 °C, 5% CO2, 95% humidity. The cell nuclei were stained with Hoechst 34580. The movie was created with ImageJ/Fiji (version 1.54d).

<https://drive.google.com/file/d/1MBL_qX1VbI18Q4Bh8l5MSUQeWZButpox/view?usp=drive_link>

**Supplementary Movie S3: Visualization of infection in whole mouse brain.** The mouse was infected intracranially with tGFP-TBEV. After the first symptoms occurred, the mouse was humanely killed, and the brain was dissected. The brain was fixed and cleared using the FDISCO method. The light-sheet microscope was used for imaging and the movie was generated using Imaris v10.0.0. (Oxford Instruments).

<https://drive.google.com/file/d/1wt3-UxjV0qwfMMGgvGhaXTgyYcIIa6qA/view?usp=drive_link>


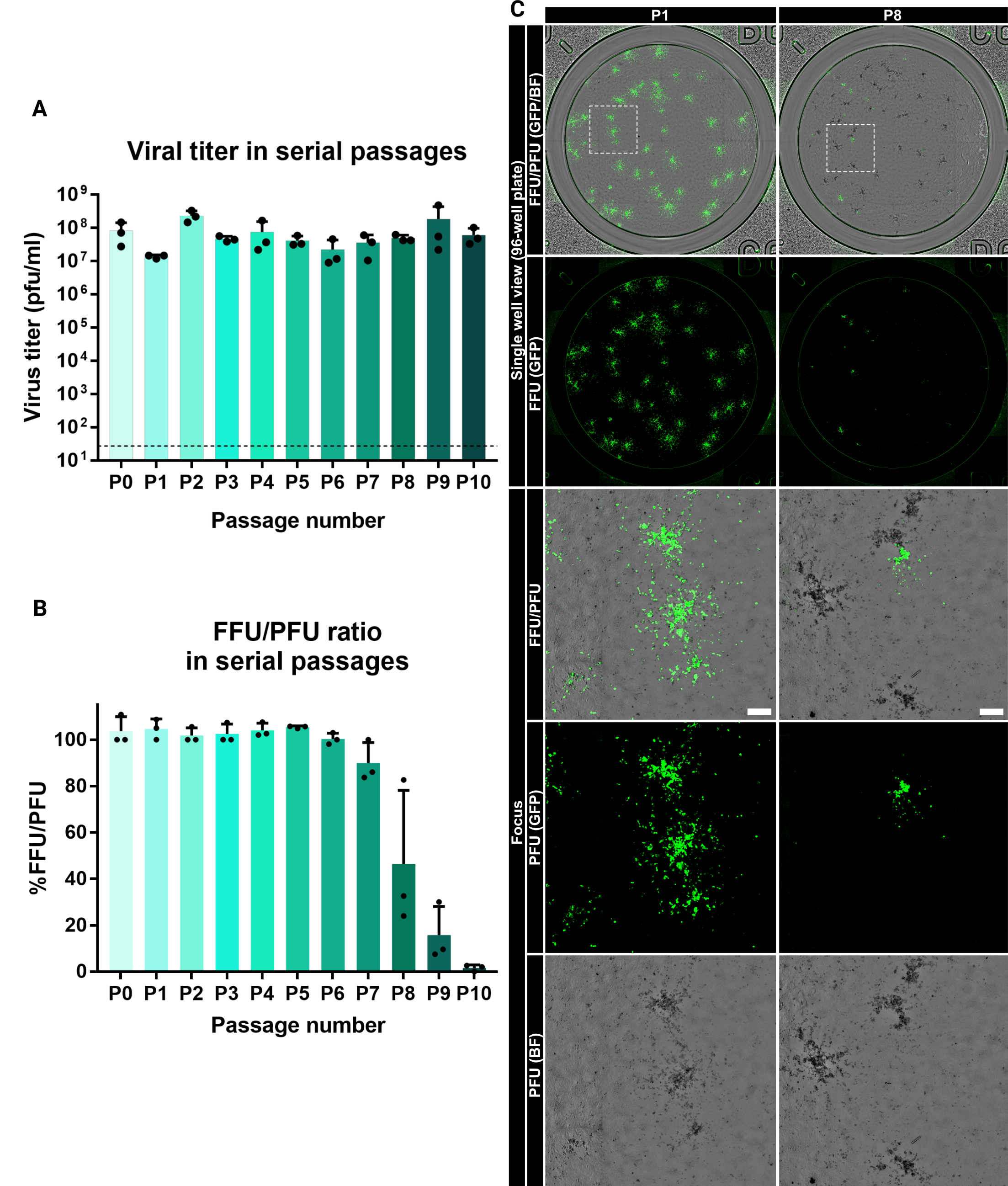


**Supplementary Figure 1: Fluorescence and viral titer determination across serial passages**. A: Viral titer was determined in each passage using plaque assay (n = 3). B: FFU/PFU ratios were calculated by imaging and comparing fluorescent focus units (FFU) and plaque-forming units (PFU) in each passage (n = 3). C: Representative images showing FFU and PFU in passage 1 (P1) and passage 8 (P8). Scale bar = 100 μm.


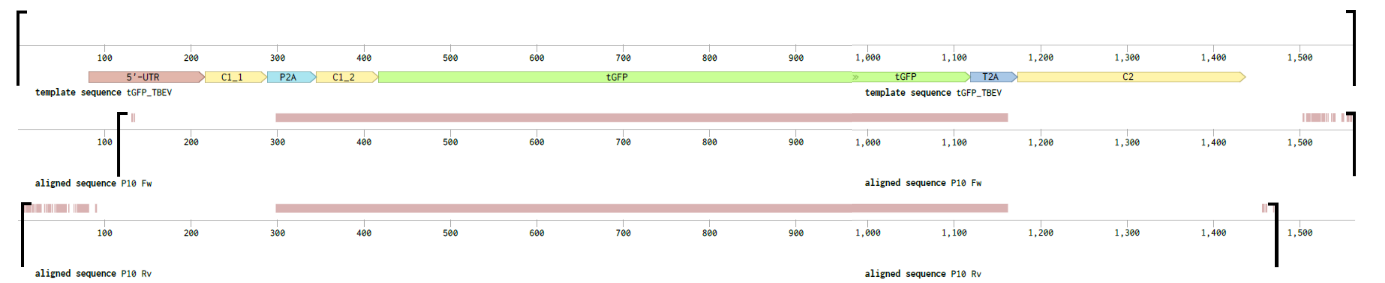


**Supplementary Figure 2: Visualization of the location of tGFP deletion in samples from P10.** Aligned sequencing results of the amplicon, generated using primers 5UTR_Fw and prM_Rv, illustrate the location of the tGFP deletion in tGFP-TBEV P10. The deletion resulted from homologous recombination between the ribosome-skipping sequences P2A and T2A.


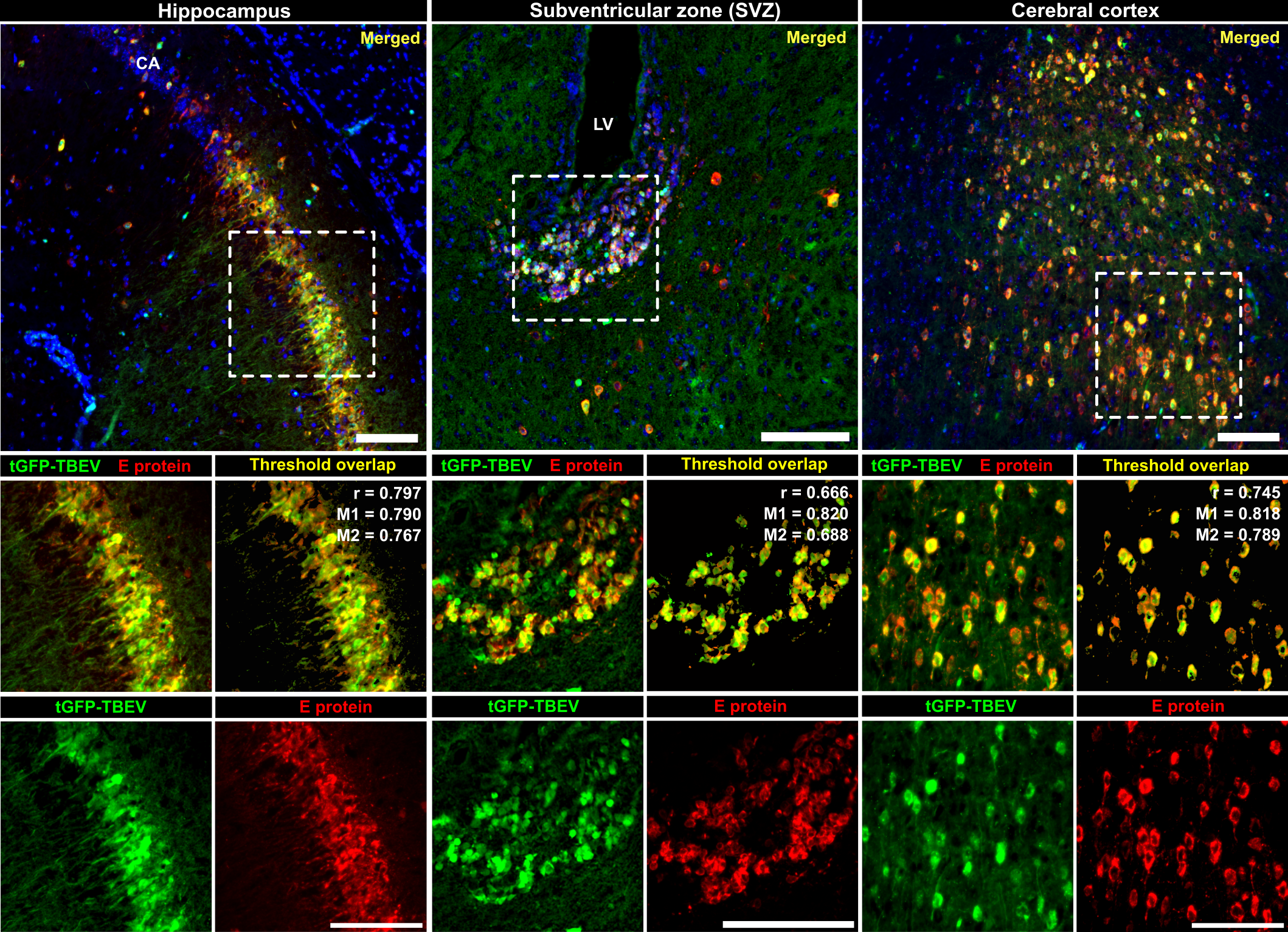


**Supplementary Figure 3: Fluorescent imaging of immunostained mouse brain slices from tGFP-TBEV-infected mice showing co-localization with E protein.** Brain sections were immunostained with anti-E protein (red) to visualize flaviviral infection. Co-localization with the tGFP signal (green) was analysed. White frames indicate the zoomed-in area. Anatomical landmarks are labelled: LV = lateral ventricle, CA = cornus ammoni. Scale bar = 100 μm. Pearson’s correlation coefficient (*r*), as well as thresholded Mander’s overlap coefficients M1 (green overlapping red) and M2 (red overlapping green), are indicated. "Threshold overlap" refers to the overlap between thresholded regions used for calculating M1 and M2. Representative images are shown (n = 5).


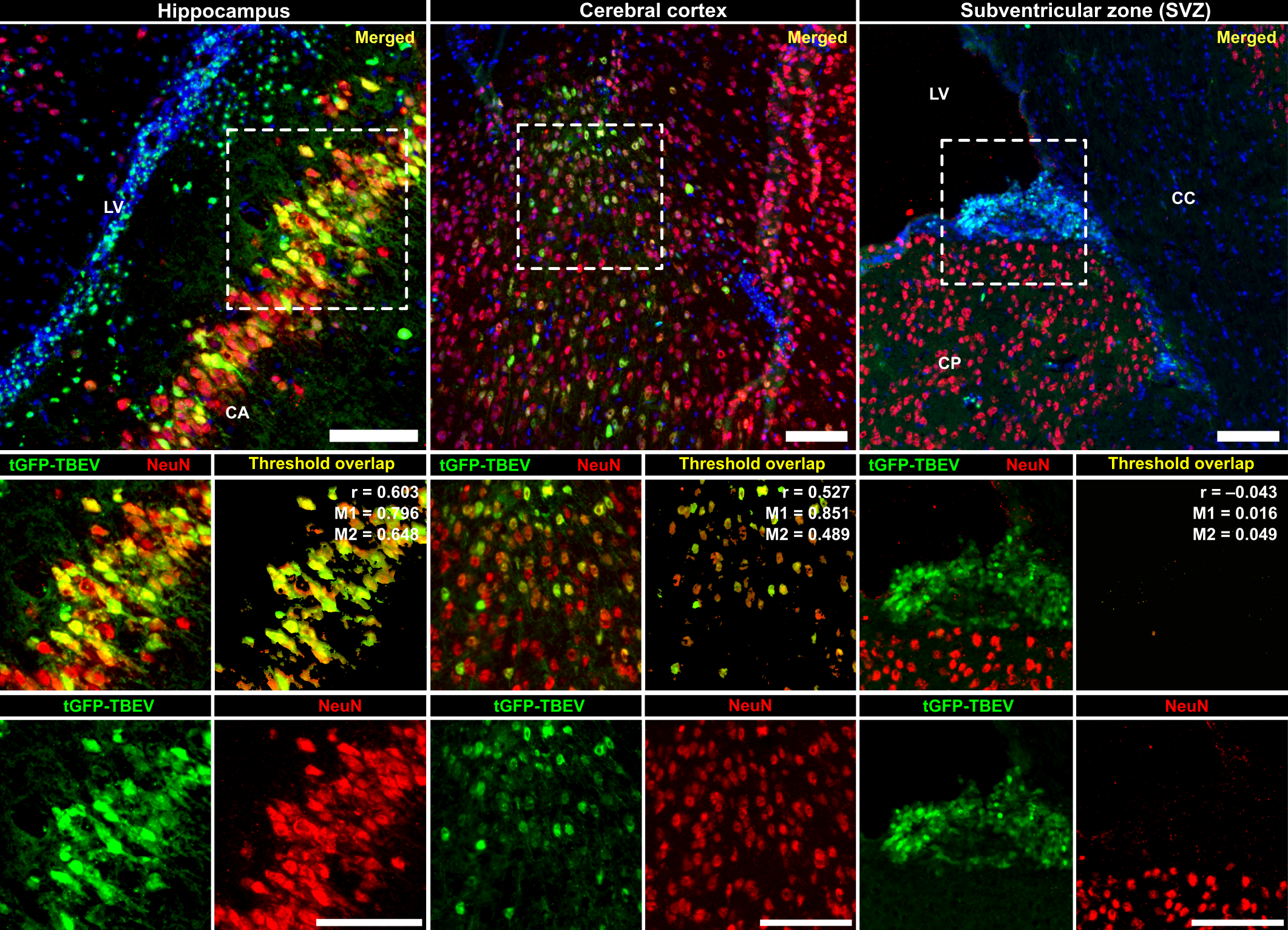


**Supplementary Figure 4: Fluorescent imaging of immunostained mouse brain slices from tGFP-TBEV-infected mice showing co-localization with mature neurons.** Brain sections were immunostained with anti-NeuN (red) to visualize mature neurons. Co-localization with the tGFP signal (green) was analysed. White frames indicate the zoomed-in area. Anatomical landmarks are labelled: LV = lateral ventricle, CA = cornus ammoni, CC = corpus callosum, CP = caudoputamen. Scale bar = 100 μm. Pearson’s correlation coefficient (*r*), as well as thresholded Mander’s overlap coefficients M1 (green overlapping red) and M2 (red overlapping green), are indicated. "Threshold overlap" refers to the overlap between thresholded regions used for calculating M1 and M2. Representative images are shown (n = 5).


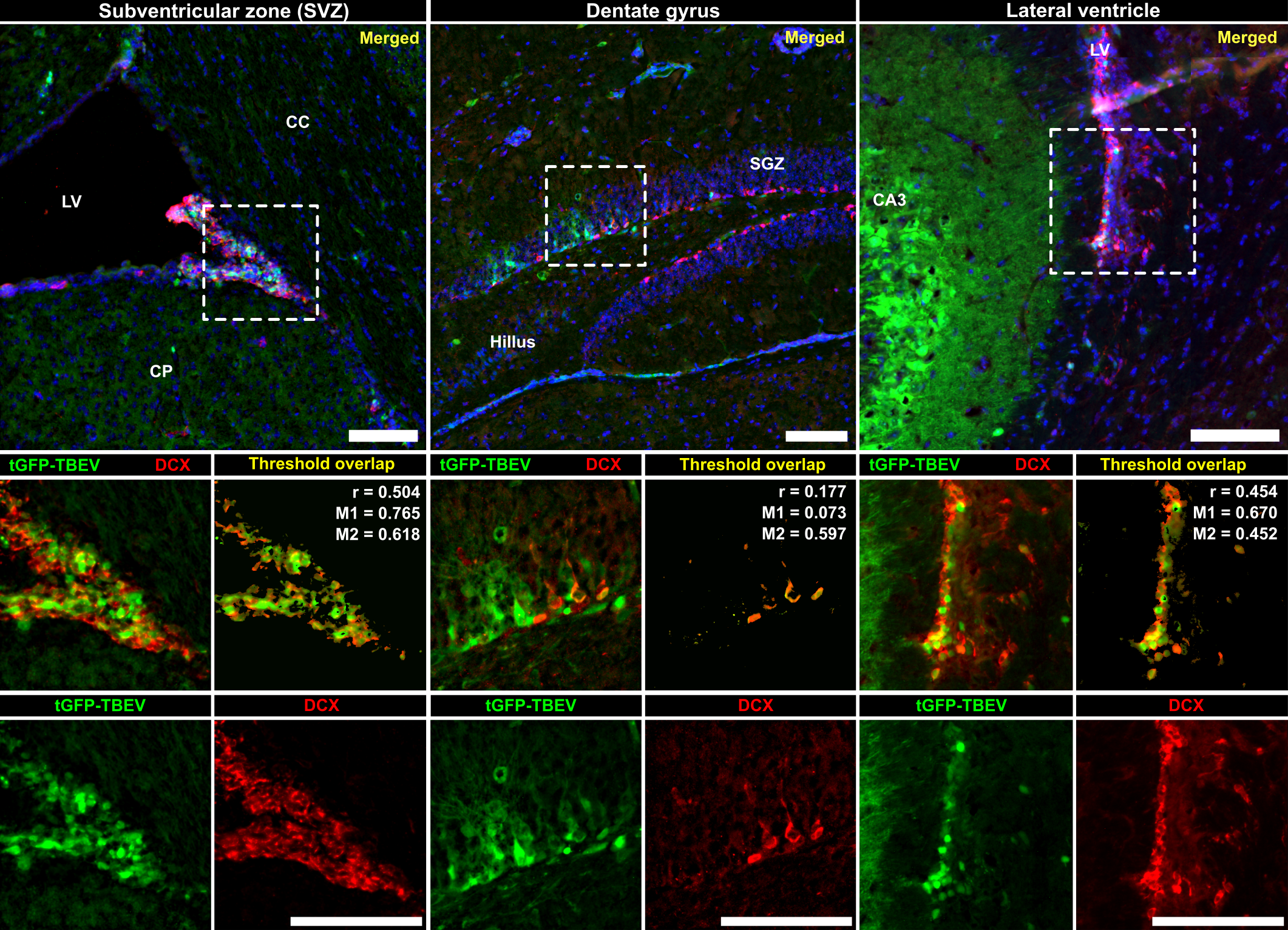


**Supplementary Figure 5: Fluorescent imaging of immunostained mouse brain slices from tGFP-TBEV-infected mice showing co-localization with immature neurons.** Brain sections were immunostained with anti-DCX (red) to visualize immature neurons. Co-localization with the tGFP signal (green) was analysed. White frames indicate the zoomed-in area. Anatomical landmarks are labelled: LV = lateral ventricle, CC = corpus callosum, CP = caudoputamen, SGZ = subgranular zone, CA3 = cornus ammoni 3 – specific area of the hippocampus. Scale bar = 100 μm. Pearson’s correlation coefficient (*r*), as well as thresholded Mander’s overlap coefficients M1 (green overlapping red) and M2 (red overlapping green), are indicated. "Threshold overlap" refers to the overlap between thresholded regions used for calculating M1 and M2. Representative images are shown (n of the brains analysed = 5).


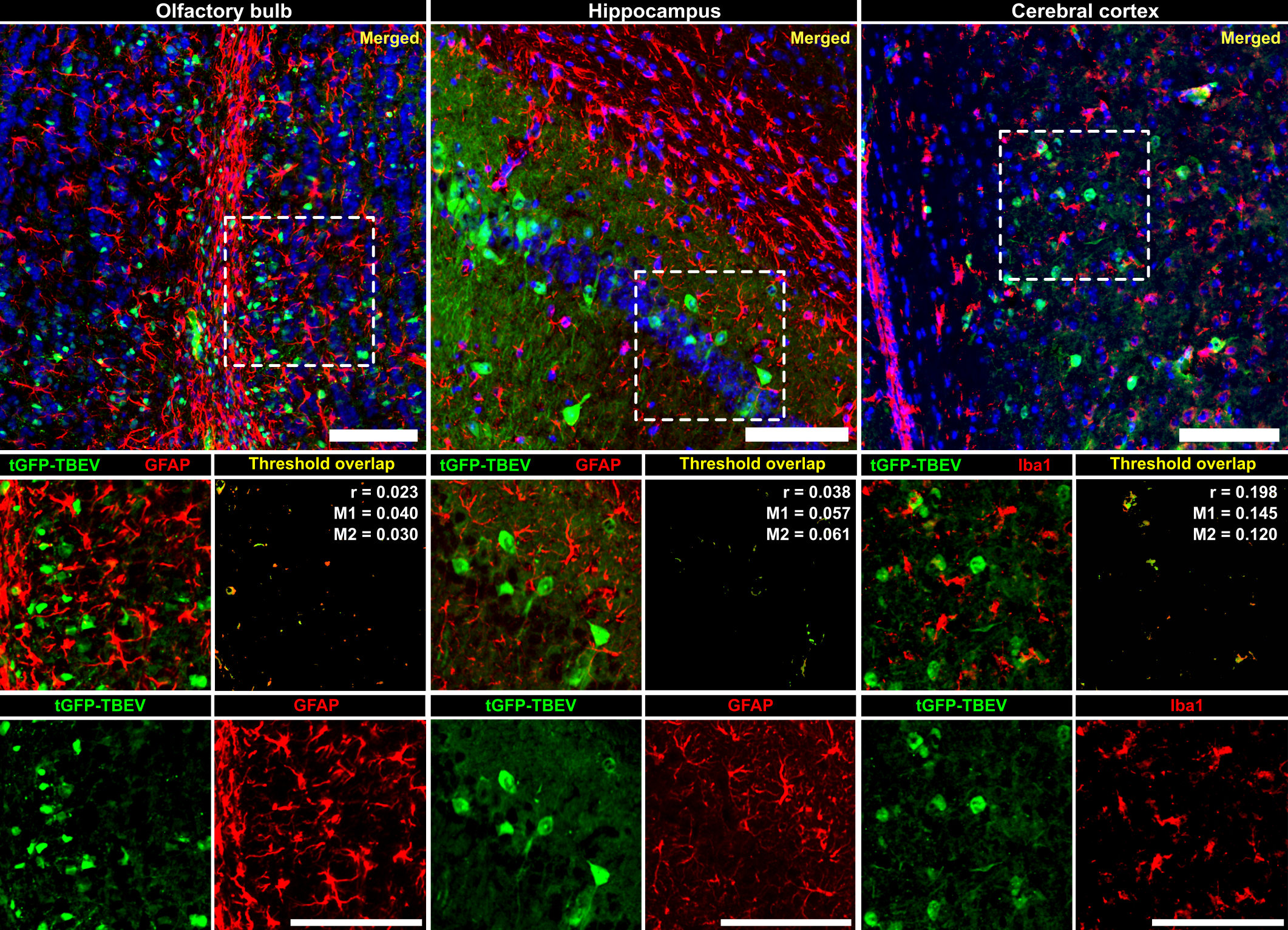


**Supplementary Figure 6: Fluorescent imaging of immunostained mouse brain slices from tGFP-TBEV-infected mice showing no co-localization with microglia or astrocytes.** Brain sections were immunostained with anti-Iba1 (red) or anti-GFAP (red) to visualize microglia or astrocytes. Co-localization with the tGFP signal (green) was analysed. White frames indicate the zoomed-in area. Scale bar = 100 μm. Pearson’s correlation coefficient (*r*), as well as thresholded Mander’s overlap coefficients M1 (green overlapping red) and M2 (red overlapping green), are indicated. "Threshold overlap" refers to the overlap between thresholded regions used for calculating M1 and M2. Representative images are shown (n of the brains analysed = 5).

**
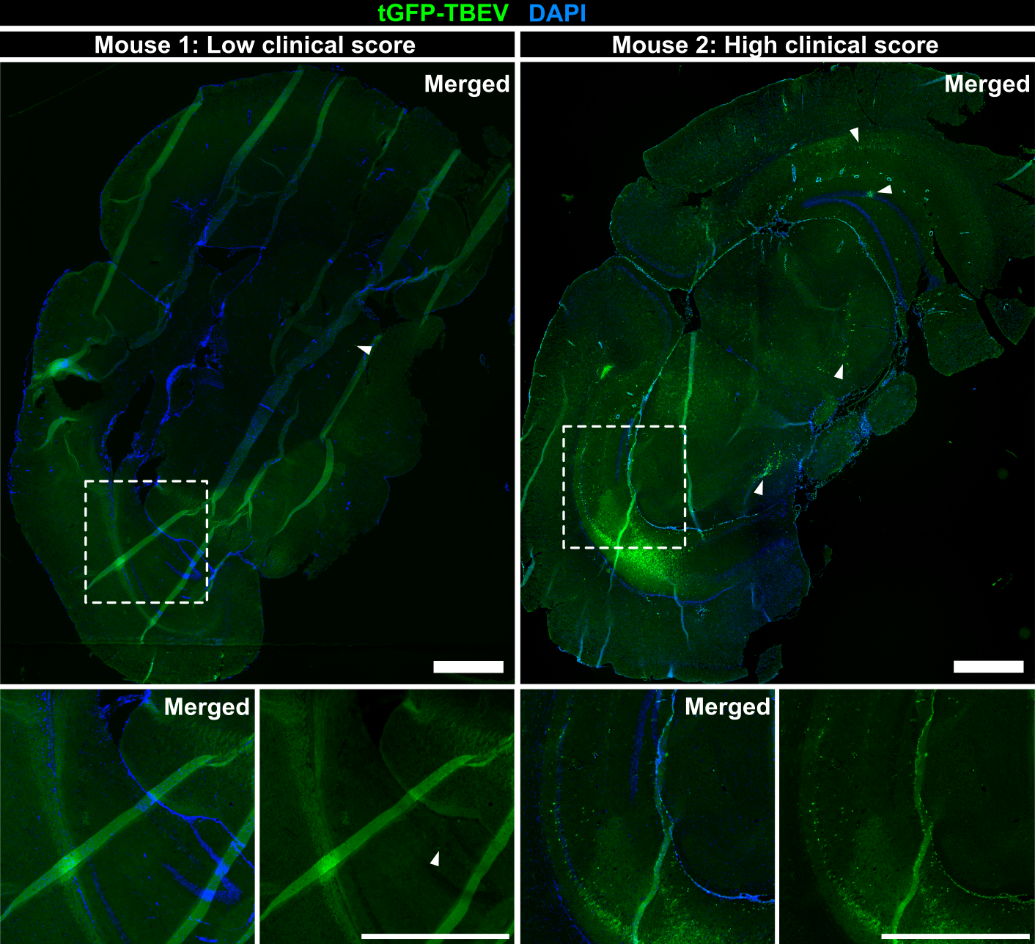
**

**Supplementary Figure 7:** **Coronal sections of mouse brains from two animals at different stages of infection.** The Mouse 1 was euthanized at the beginning of the clinical manifestation of the infection, showing a limited specific fluorescent signal (few fluorescent neurons indicated by arrows). In contrast, the second animal – Mouse 2, euthanized at a more advanced stage of the disease, exhibited numerous loci of fluorescent cells, mainly in the hippocampal region. Scale bar = 1000 µm.

**Supplementary Table 1: List of primers**

| **Primer name** | **Primer sequence** | **Localization** | **Localization in the TBEV genome** | **Amplicon lenght** |
| --- | --- | --- | --- | --- |
| **tGFP_Fw** | AGGACAGCGTGATCTTCACC | tGFP | - | 418 bp |
| **C2_Rv** | CGCATCAACACGAGCCCATT | C | 254 - 235 |  |
| **5UTR_Fw** | AGATTTTCTTGCACGT | 5’ UTR | 1-16 | 1417 bp or 532 bp |
| **prM_Rv** | TTCCTTCAGCTCTGATC | PrM/M | 532-516 |  |

**Supplementary Table 2: List of antibodies**

|  | **Target** | **Host** | **Produced by** | **Used dilution** |
| --- | --- | --- | --- | --- |
| **Primary antibodies** | | | | |
| **4G2 (mouse experiments)** | Flaviviral E protein | Mouse | Sigma-Aldrich  ZMS1070 | 1:250 |
| **4G2 (BCS experiments)** | Flaviviral E protein | Hybridoma | Alves Group, AB-229915 | 1:10 |
| **T034** | Flaviviral E protein | Hybridoma | (Agudelo et al., 2021) | 1:500 |
| **NeuN polyclonal Antibody** | Mouse Fox-3 (mature neurons) | Rabbit | Invitrogen  PA5-78499 | 1:500 |
| **Recombinant Anti-Calbindin antibody** | Mouse Calbindin (Purkinje cells) | Rabbit | Abcam  [EPR22698-236] | 1:500 |
| **Doublecortin Polyclonal Antibody** | Doblecortin (immature neruons) | Rabbit | Invitrogen  48-1200 | 1:500 |
| **IBA1 Polyclonal Antibody** | Iba1 (microglia) | Rabbit | Invitrogen  PA5-27436 | 1:100 |
| **GFAP Polyclonal Antibody** | GFAP | Rabbit | Invitrogen  PA5-16291 | 1:500 |
| **Secondary antibodies** | | | | |
| **Alexa FluorTM 647** | Mouse IgG | Goat | Invitrogen A-21235 | 1:1000 |
|  | Human IgG |  |  |  |
|  | Rabbit IgG |  |  |  |
| **CyTM3 AffiniPureTM Donkey Anti-Mouse IgG** | Mouse IgG | Donkey | Jackson ImmunoResearch  A-212245  715-165-151 | 1:1000 |
